# Root Endophytic Microorganisms Contribute to the Attribute of Full-Year Shooting in Woody Bamboo *Cephalostachyum pingbianense*

**DOI:** 10.3390/microorganisms12091927

**Published:** 2024-09-22

**Authors:** Lushuang Li, Bin Li, Qing Li, Lianchun Wang, Hanqi Yang

**Affiliations:** 1 Key Laboratory of Forest Resources Conservation and Utilization in the Southwest Mountains of China, Ministry of Education, Southwest Forestry University, Kunming 650224, China; lilushuang@126.com; 2 Forestry College, Southwest Forestry University, Kunming 650224, China; 3 Horticultural Research Institute, Yunnan Academy of Agricultural Sciences, Kunming 650205, China; libin2015caf@126.com; 4 Yunnan Forestry Double Center, Yunnan Forestry and Grassland Bureau, Kunming 650051, China; ynslyszx2020@yeah.net; 5 Institute of Highland Forest Science, Chinese Academy of Forestry, Kunming 650233, China; 6 Key Laboratory of Breeding and Utilization of Resource Insects, National Forestry and Grassland Administration, Kunming 650233, China

**Keywords:** bamboo shooting, plant endophyte, 16S rRNA and ITS rDNA sequencing, microbial function, seasonal variation

## Abstract

*Cephalostachyum pingbianense* (Hsueh & Y.M. Yang ex Yi et al.) D.Z. Li & H.Q. Yang is unique among bamboo species for its ability to produce bamboo shoots in all seasons under natural conditions. Apart from the physiological mechanism, information regarding the effects of endophytic microorganisms on this full-year shooting characteristic is limited. We hypothesize that root endophytic microorganisms will have a positive impact on the full-year bamboo shooting characteristic of *C. pingbianense* by increasing the availability or supply of nutrients. To identify the seasonal variations in the root endophytic bacterial and fungal communities of *C. pingbianense,* and to assess their correlation with bamboo shoot productivity, the roots of *C. pingbianense* were selected as research materials, and the 16S rRNA and ITS rDNA genes of root endophytic microorganisms were sequenced using the Illumina platform. Following this sequencing, raw sequencing reads were processed, and OTUs were annotated. Alpha and beta diversity, microbial composition, and functional predictions were analyzed, with correlations to bamboo shoot numbers assessed. The results showed that seasonal changes significantly affected the community diversity and structure of root endophytic microbes of *C. pingbianense*. Bacterial communities in root samples from all seasons contained more nitrogen-fixing microorganisms, with members of the Burkholderiales and Rhizobiales predominating. The relative abundances of ectomycorrhizal and arbuscular mycorrhizal fungi in the autumn sample were significantly higher than in other seasons. Correlation analysis revealed that the bamboo shoot productivity was significantly and positively correlated with bacterial functions of nitrogen fixation, arsenate detoxification, and ureolysis, as well as with symbiotrophic fungi, ectomycorrhizal fungi, and arbuscular mycorrhizal fungi. At the genus level, the bacterial genus *Herbaspirillum* and the fungal genera *Russula*, unclassified_f_Acaulosporaceae, and unclassified_f_Glomeraceae were found to have a significant positive correlation with bamboo shoot number. Our study provides an ecological perspective for understanding the highly productive attribute of *C. pingbianense* and offers new insights into the forest management of woody bamboos.

## 1. Introduction

Plant endophytes are microorganisms that live asymptomatically within plants at various stages of their life cycles and are integral to plant biology. While many endophytes’ roles are not fully understood, some are known to significantly influence plant growth, development, adaptability, and diversification. Generally, endophytes affect the overall health and productivity of plants by facilitating access to nutrients [1], protecting plants from pathogens [2], increasing tolerance of plants to stress [3], and producing plant hormones [4]. Studies indicate that plant endophytes can serve as biocontrol agents to meet the needs of environmental protection, as well as to improve crop quality and yield, which have great potential in sustainable agriculture [5,6,7].

Bamboo, recognized for its rapid growth, rich biomass, high regenerative capacity, and versatility, is a potential sustainable forest resource. Currently, researchers have studied the endophytic bacteria or fungi of several bamboo species, including Moso bamboo (*Phyllostachys edulis*), Lei bamboo (*Phyllostachys praecox*), *Ampelocalamus luodianensis*, *Bambusa rigida*, *Pleioblastus amarus* and so on [8,9,10,11]. It has been found that certain species of *Alcaligenes*, *Enterobacter*, and *Bacillus* screened from the root, rhizome, stem, and leaves of Moso bamboo had the function of phosphorus and potassium dissolution. After inoculating their compound bacterial suspension, the shooting duration of bamboo shoots in the following spring—defined as the time from the start to the end of emergence—was prolonged [12,13]. A novel *Paraburkholderia* strain was isolated from Moso bamboo roots, and gene homologs encoding biosynthesis of the plant growth-promoting chemicals acetoin and 2,3-butanediol were identified in the genome of this strain. In laboratory conditions, it was able to promote the growth of Moso bamboo and seedlings of *Arabidopsis thaliana* [14].

The diversity and composition of bamboo endophytic microbial communities are influenced by tissue type, growth stage, and management practices. In Moso bamboo, the composition of the endophytic bacterial community differed between bamboo shoots and stems, with bacterial species in stems increasing as the plant grows [15]. In *Drepanostachyum luodianense*, different tissues harbored rich endophytic fungi, and the Shannon index of fungal communities showed a gradually increasing trend from leaves to stems to roots [9]. The diversity, composition, and function of endophytic microbial communities in the roots of Lei bamboo were significantly influenced by intensive management [10]. Climate and seasonal changes have been shown to affect endophytic communities in various plants [16,17], but to date, the impact of seasonal variations on bamboo endophytes remains largely unexplored. Gaining insight into the seasonal changes of bamboo endophytes may shed light on their potential functions in host growth and health.

As a typical clonal plant, bamboo mainly relies on shoots for asexual reproduction, making the bamboo shooting period crucial in its life cycle. In general, monopodial bamboos like *Phyllostachys* shoot in the spring and last for 1~2 months [18,19]. Sympodial bamboos like *Dendrocalamus* shoot in the summer and autumn, with a shooting period of 1~3 months [20,21]. The shooting onset and duration of most amphipodial bamboos (e.g., *Pseudosasa*) fall between those of monopodial and sympodial bamboos [22]. While the shooting duration varies by species, most bamboo species end the shooting process by early November. The object of this study, *Cephalostachyum pingbianense* (Hsueh & Y.M. Yang ex Yi et al.) D.Z. Li & H.Q. Yang, is a small-scaled sympodial bamboo that naturally distributes under humid evergreen broad-leaved forests in southeastern Yunnan [23]. It attracted our attention because it is currently the only bamboo species known to produce bamboo shoots in four seasons under natural conditions. Compared with ordinary bamboos that shoot in specific seasons, *C. pingbianense* represents a unique shooting type or mechanism. Considering the year-round shoot production of *C. pingbianense*, the sprouting of bamboo shoots and the growth of young culms theoretically necessitate a substantial nutrient supply. It remains an open question whether microorganisms contribute to this nutrient demand, making it a subject worthy of further investigation.

While some studies have examined microorganisms associated with bamboo plants, few have explored the correlation between bamboo shoot productivity and endophytic microorganisms. In previous studies, we have investigated the molecular and physiological regulatory mechanisms of the full-year shooting characteristic in *C. pingbianense* [24,25]. However, the potential influence of bamboo endophytic microorganisms on this characteristic remains unknown. In light of the various positive roles that microorganisms play in plant growth, we speculate that they may have a beneficial effect on this specific trait. Since roots are one of the main habitats of endophytic bacteria and fungi [26], this study sequenced the 16S rRNA and ITS rDNA genes of root endophytic microorganisms of *C. pingbianense* across different seasons. Our objectives were to detect the seasonal variation of root endophytic bacterial and fungal communities of *C. pingbianense* and to assess the potential correlation between bamboo shoot productivity and root endophytic microorganisms. The results will enhance our understanding of the role of endophytic microbes in regulating bamboo shoot productivity and provide new ecological insights into the management of woody bamboo forests.

## 2. Materials and Methods

### 2.1. Root Sample Collection and Surface Sterilization

Bamboo root samples of *C. pingbianense* were collected from Dawei Mountain National Nature Reserve (DMNNR, Lat. 22°54′ N, 103°42′ E), Yunnan Province, China. According to the Köppen’s climate classification, the region experiences a tropical monsoon climate, with the rainy season lasting from May to October. The sampling site is situated at an altitude of approximately 2000 m, with an average annual temperature of 16.5 °C, and an average annual precipitation of 1649 mm. Field sampling at Dawei Mountain was approved by the Pingbian Sub-bureau of DMNNR. Three 30 × 30 m sampling plots were established as replicates within the central distribution area of *C. pingbianense*. Each plot contained five subplots: one large subplot of 8 × 8 m and four smaller subplots of 5 × 5 m, each separated by at least 3 m. Ten vigorous bamboo clusters were labeled within the 8 × 8 m subplot, resulting in a total of 30 marked clusters across the three replicates for assessing bamboo shoot emergence. The four smaller subplots were designated for seasonal root sampling, with collections occurring in July 2020 (summer), October 2020 (autumn), January 2021 (winter), and April 2021 (spring). For each sampling, after digging out the bamboo rhizome, healthy and fresh roots were selected and their surfaces were washed with running tap water. The roots were then sequentially immersed in sterile water (30 s), 70% ethanol (3 min), 2.5% NaClO (5 min), and 70% ethanol (30 s), followed by rinsing three times with sterile water. The last washing solution was applied to the surface of Luria-Bertani (LB) and Potato Dextrose Agar (PDA) media, and the root surface was considered thoroughly sterilized if no colonies emerged after culture. These sterilized roots were cut into small sections and stored at −70 °C for DNA extraction. When conducting the investigation of the number of emerging shoots, newly emerged bamboo shoots were marked and numbered to distinguish them from those emerging later. Shoot emergence was defined as the tip protruding 1–2 cm above the ground. Moreover, the emerged bamboo shoot numbers of the marked bamboos in July 2020, October 2020, January 2021, and April 2021 were recorded as bamboo shooting data of *C. pingbianense* in four seasons, as detailed in a previous study [27] (Table S1).

### 2.2. DNA Extraction and Illumina Sequencing

The genomic DNA of bamboo root samples was extracted using the plant DNA isolation reagent kit (Takara, TaKaRa Bio, Beijing, China). In this process, each sample used 0.5g of root tissue. DNA quality was examined using 1% agarose gel electrophoresis and the NanoDrop 2000 spectrophotometer (Thermo Fisher Scientific, Wilmington, DE, USA). To identify the characteristics of root endophytic bacterial communities, the V5–V7 variable region of the 16S rRNA gene was amplified. The primers for the first round of PCR were 799F (5′-AACMGGATTAGATACCCKG-3′) and 1392R (5′-ACGGGCGGTGTGTRC-3′), and for the second round were 799F (5′-AACMGGATTAGATACCCKG-3′) and 1193R (5′-ACGTCATCCCCACCTTCC-3′). The reason for performing two rounds of PCR was to remove contamination of chloroplasts and mitochondria. In addition, the primer set ITS1F (5′-CTTGGTCATTTAGAGGAAGTAA-3′) and ITS2R (5′-GCTGCGTTCTTCATCGATGC-3′), targeting the internal transcribed spacer 1 (ITS1) region of the ribosomal RNA gene, was used to classify fungi [28]. DNA sequencing was conducted on the Illumina MiSeqPE300 Platform (Illumina, San Diego, CA, USA). PCR amplification and subsequent DNA sequencing followed the standard protocol of Majorbio Bio-pharm Technology Co., Ltd. (Shanghai, China), as described in previous studies [29,30]. The raw sequencing data have been uploaded to the NCBI Sequence Read Archive (SRA) under the BioProject number PRJNA1117650.

### 2.3. Data Processing and Analysis

Raw sequencing reads were processed for splicing and quality control using FLASH (v1.2.11, Johns Hopkins University, Baltimore, MD, USA) [31] and fastp (v0.19.6, HaploX Biotechnology Co., Ltd, Shenzhen, China) software [32]. Operational taxonomic units (OTUs) were clustered using the UPARSE algorithm with 97% sequence similarity [33], and representative OTUs with the highest frequency were selected for further annotation. The Silva database (Release 138, https://www.arb-silva.de, accessed on 16 December 2022) and Unite database (Release 8.0, https://unite.ut.ee/index.php, accessed on 16 December 2022) were used for bacterial and fungal data annotation, respectively.

To ensure the uniformity of the sequences of the samples so that they could be analyzed under the same criterion subsequently, we rarified the bacterial OTU tables to the minimum reads per sample, with the same approach applied to fungal OTU tables. Chao1 and Shannon indexes were used to characterize the alpha diversity of the root endophytic microbial community, and a one-way analysis of variance (ANOVA) was conducted to assess whether there were any statistically significant differences in diversity indexes among samples from the four seasons. Beta diversity of the microbial community was analyzed using principal coordinate analysis (PCoA) based on the Bray-Curtis distance algorithm, while within- and between-group similarity was compared using an analysis of similarities (ANOSIM) test based on the same algorithm. Both of these two analyses were performed with the vegan package of the R software (v 3.3.1).

The composition and relative abundance of endophytic microorganisms in the samples of each season were calculated at the phylum, order, and genus levels. Venn diagrams illustrated common or unique OTUs among samples from different seasons. Linear discriminant analysis (LDA) effect size (LEfSe) was applied to identify taxa with significant differences among samples [34]. Cladograms were generated using the LEfSe algorithm via the Huttenhower Galaxy web application (Huttenhower Lab, Boston, MA, USA; http://huttenhower.sph.harvard.edu/galaxy, accessed on 9 February 2023), with the LDA threshold set to 3.5 for bacteria and 3 for fungi.

The ecological functions of bacterial endophytic microorganisms were predicted using the FAPROTAX software (v1.2.1, University of Oregon, Eugene, OR, USA) [35], while functional profiling of fungal communities was predicted based on FUNGuild database (http://www.funguild.org/, accessed on 17 April 2023) [36]. Using the bamboo shoot numbers from different seasons and the microbial functions or key genera in corresponding seasons as variables, Spearman’s correlation analysis was performed using SPSS 24.0 software (SPSS Inc., Chicago, IL, USA).

## 3. Results

### 3.1. Community Diversities of Bacterial and Fungal Root Endophytes in C. pingbianense across Different Seasons

After sequencing, a total of 180,255 valid sequences of 16S rRNA genes were obtained from 12 samples, resulting in 1461 bacterial OTUs through OTU clustering at 97% sequence similarity. In addition, 654,620 effective sequences of ITS rRNA genes yielded 1739 fungal OTUs. The coverage indexes of the bacterial and fungal communities in all samples were higher than 98%, indicating that the sequencing data were sufficient and could reflect the diversity of endophytic communities in the samples (Table S2).

**Figure 1:**
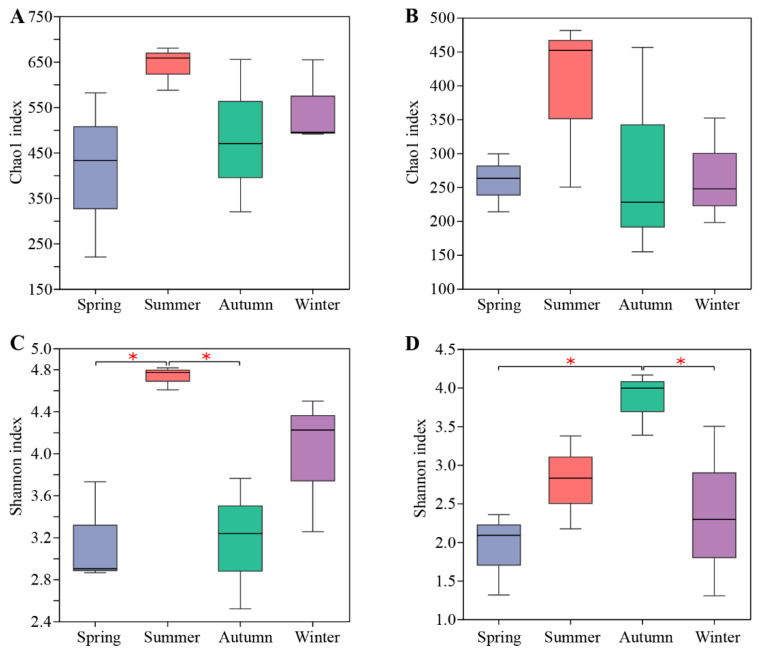
Alpha diversity of endophytic bacterial and fungal communities in root samples of *C. pingbianense* in different seasons. (**A**,**B**) Chao1 indexes of bacterial (**A**) and fungal (**B**) communities. (**C**,**D**) Shannon indexes of bacterial (**C**) and fungal (**D**) communities. * Signiffcant differences at *p* < 0.05 level.

By comparing the richness and diversity indexes of root endophytic microbial communities in different seasons, we observed that seasonal changes significantly affected the community diversities of bacterial and fungal root endophytes of *C. pingbianense*. The endophytic bacterial Shannon indexes in root samples from the four seasons were ranked in descending order as summer, winter, autumn, and spring, with the difference between the summer and autumn sample, and that between the summer and spring sample, reaching a significant level (*p* < 0.05) (Figure 1C). The Chao1 index of the bacterial community exhibited a similar trend to the Shannon index across the seasons but did not show significant differences among the samples from the four seasons (Figure 1A). No significant differences were found in the fungal Chao1 index among the seasonal samples (Figure 1B). However, the fungal Shannon index in the autumn sample was significantly higher than that in the spring and winter samples (*p* < 0.05), with the Shannon indexes in the four seasons showing a descending trend of autumn, summer, winter, and spring (Figure 1D).

**Figure 2:**
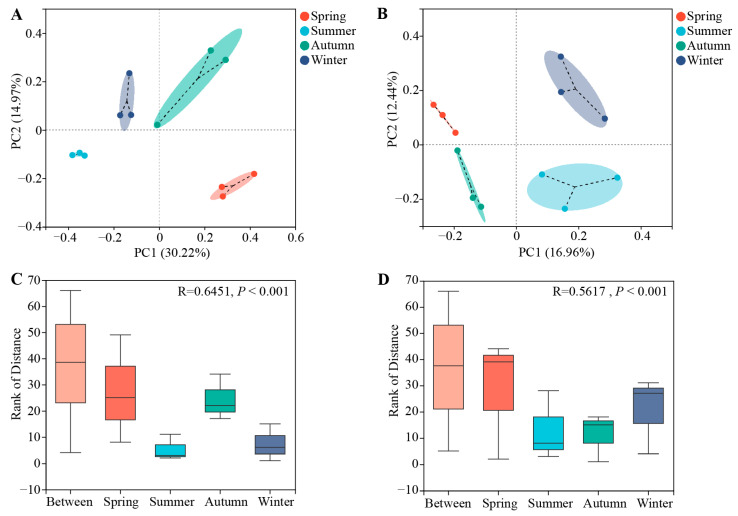
Beta diversity and analysis of similarities (ANOSIM) of endophytic bacterial and fungal communities in root samples of *C. pingbianense* in different seasons. (**A**,**B**) Principal coordinate analysis (PCoA) of bacterial (**A**) and fungal (**B**) communities. (**C**,**D**) ANOSIM based on the Bray-Curtis distance algorithm of bacterial (**C**) and fungal (**D**) communities.

The results of the PCoA analysis demonstrated a clear separation of root endophytic bacterial communities across different seasons, and the same was true for the fungal communities. (Figure 2A,B). ANOSIM analysis further confirmed significant differences in bacterial community structure among the seasonal samples (r = 0.6451, *p* = 0.001) (Figure 2C), as well as in the fungal community structure (r = 0.5617, *p* = 0.001) (Figure 2D). These results suggest that seasonal variation is one of the driving factors for changes in the community structure of root endophytic bacteria and fungi in *C. pingbianense*.

### 3.2. Community Composition of Bacterial and Fungal Root Endophytes in C. pingbianense across Different Seasons

**Figure 3:**
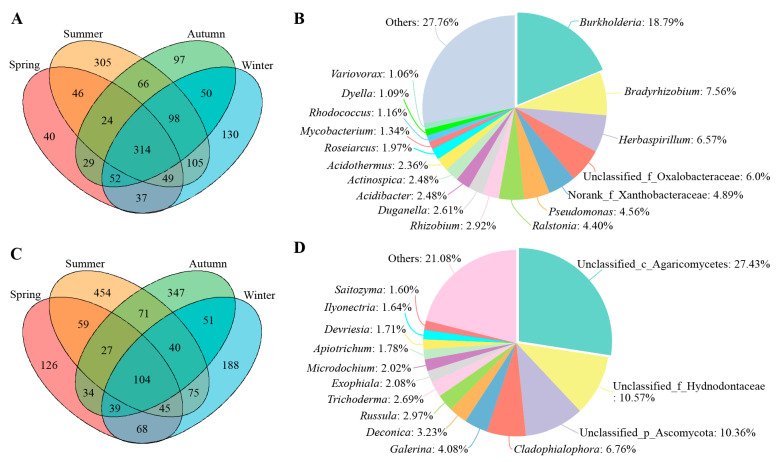
Shared endophytic microbial groups in root samples of *C. pingbianense* in the four seasons. (**A**,**C**) Venn diagrams showing the number of shared and unique bacterial (**A**) and fungal (**C**) OTUs among samples. (**B**,**D**) Shared bacterial (**B**) and fungal (**D**) genera among samples.

Venn diagrams showed that there were 314 shared bacterial OTUs among the root samples across the four seasons, accounting for 31.18~53.13% of the total bacterial OTUs in each season (Figure 3A). The shared fungal OTUs numbered 104, representing 11.89~20.72% of the total fungal OTUs in each season (Figure 3C). The annotation results of common OTUs revealed that shared bacterial genera primarily included *Burkholderia* (18.79%), *Bradyrhizobium* (7.56%), *Herbaspirillum* (6.57%), norank_f_Xanthobacteraceae (4.89%), and *Pseudomonas* (4.56%) (Figure 3B). The shared fungal taxa consisted mainly of members of Agaricomycetes or Ascomycota (Figure 3D).

**Figure 4:**
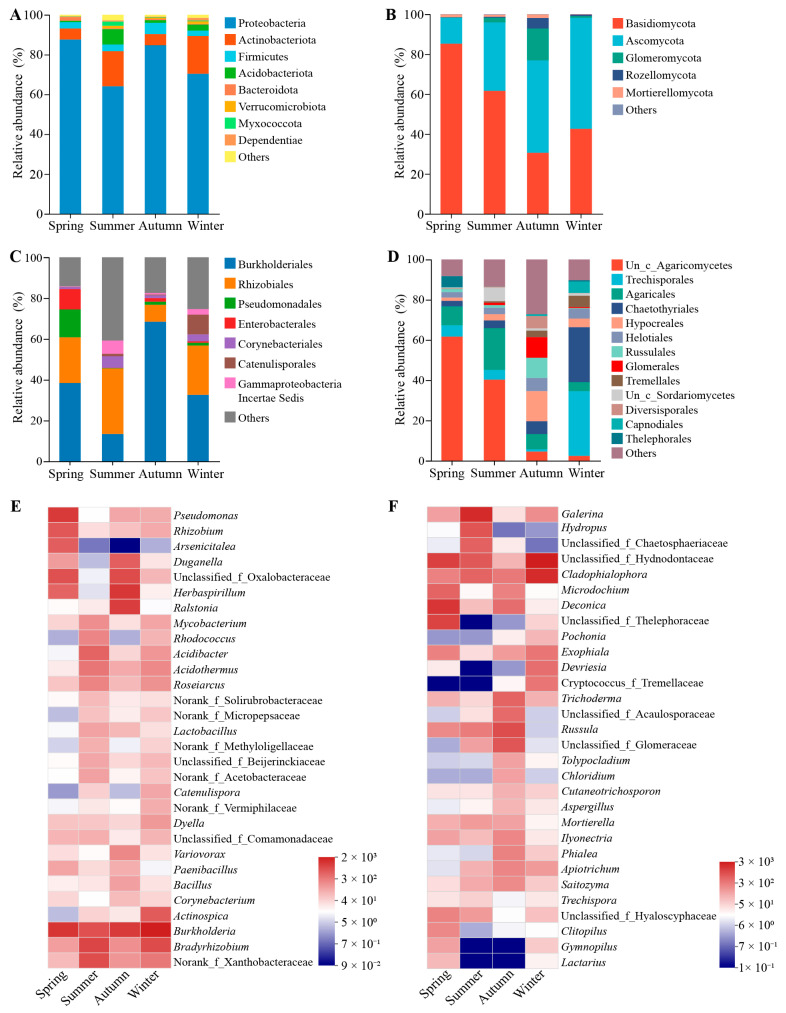
Endophytic microbial community composition in root samples of *C*. *pingbianense* in different seasons. (**A**,**B**) Relative abundances of main bacterial (**A**) and fungal (**B**) community phyla. (**C**,**D**) Relative abundances of main bacterial (**A**) and fungal (**B**) community orders. (**E**,**F**) Heatmaps showing the top 30 abundant bacterial (**C**) and fungal (**D**) genera.

The community composition of the root endophytes was analyzed at various taxonomic levels. At the phylum level, bacterial or fungal taxa with a relative abundance of less than 1% were classified as “others”. Proteobacteria, Actinobacteriota, Firmicutes, and Acidobacteriota were the dominant phyla of the root endophytic bacterial communities throughout all seasons. Among these, Proteobacteria was the most dominant phylum, accounting for more than 60% of the bacterial community in each season and over 80% in spring and autumn. The relative abundance of Actinobacteriota was higher in the summer and winter samples compared to the spring and autumn samples, while Firmicutes and Acidobacteriota had the highest relative abundance in the autumn and summer samples, respectively (Figure 4A). At the order level, microorganisms with a relative abundance of less than 5% were classified as “others”. Burkholderiales and Rhizobiales were the predominant orders in the root endophytic bacterial community of *C. pingbianense*. Compared to other seasons, the autumn sample exhibited a higher relative abundance of Burkholderiales but a lower relative abundance of Rhizobiales. The spring sample had a higher proportion of bacterial taxa from Pseudomonadales and Enterobacterales, while the winter sample had a higher proportion of bacteria belonging to Catenulisporales (Figure 4C). At the genus level, we focused on the top 30 abundant bacterial and fungal genera. The genus *Burkholderia* had a high relative abundance in the root endophytic bacterial community across all seasons. In addition, genera such as *Bradyrhizobium* and norank_f_Xanthobacteraceae were relatively more abundant in the summer and winter samples, while genera such as *Herbaspirillum* and unclassified_f_Oxalobacteraceae were more abundant in the spring and autumn samples (Figure 4E).

For fungi, Basidiomycota, Ascomycota, and Glomeromycota were the dominant fungal phyla in samples from all seasons. In the spring and summer samples, the relative abundance of Basidiomycota was higher than that of Ascomycota, while the opposite trend was observed in the autumn and winter samples. Additionally, the relative abundances of Glomeromycota and Rozellomycota in the autumn sample were much higher than those in other seasons (Figure 4B). At the order level, the dominant fungal taxa varied considerably from season to season. For instance, Hypocreales, Russulales, and Glomerales were dominant orders in the autumn sample, whereas Trechisporales and Chaetothyriales were predominant in the winter sample. The order Agaricales showed higher relative abundance in the spring and summer samples compared to those from autumn and winter (Figure 4D). At the genus level, *Cladophialophora* and unclassified_f_Hydnodontaceae were characterized by high relative abundance in all samples, with the highest abundances observed in the winter sample. Fungal genera such as *Microdochium* and *Deconica* were in a high abundance in the spring and autumn samples, while *Galerina*, *Hydropus*, and unclassified_f_Chaetosphaeriaceae showed higher abundance in the summer sample. In addition, genera of *Trichoderma*, unclassified_f_Acaulosporaceae, *Russula*, and unclassified_f_Glomeraceae exhibited higher abundance in the autumn sample (Figure 4F).

### 3.3. Taxa Characterizing Seasonal Differences within the Root Endophytic Microbiome

**Figure 5:**
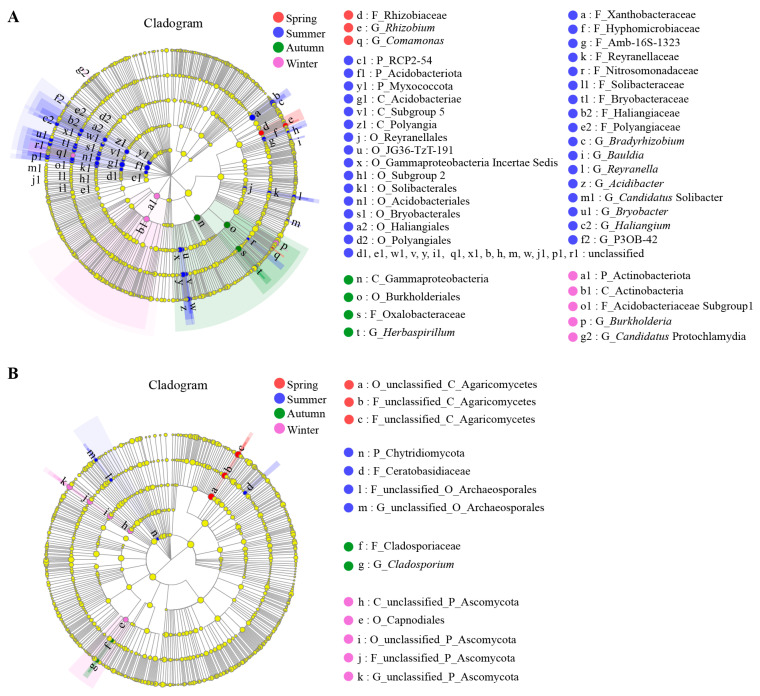
Cladograms showing taxa with different abundance values of endophytic bacterial (**A**) and fungal (**B**) communities in root samples of *C*. *pingbianense* in different seasons. The concentric circles radiating from the center of the figure represent taxonomic levels ranging from phylum to genus (phylum, class, order, family, genus). Each small circle on the different taxonomic levels represents a taxon at that level, with the size of the circle corresponding to the relative abundance. Red, blue, green, and purple circles indicate bacterial or fungal taxa that are significantly enriched and have a notable impact on intergroup differences in spring, summer, autumn, and winter, respectively. Yellow circles represent taxa that show no significant differences between groups or have no significant impact on intergroup differences.

LEfSe was employed to identify microbial biomarkers, which are taxonomic taxa with significant differences in abundance among the samples from the four seasons. The results indicated that the summer samples had the highest number of bacterial biomarkers, totaling 32 across various taxonomic levels. These included the phyla Acidobacteriota and Myxococota, the class Acidobacteriae, the orders Bryobacterales and Acidobacteriales, the families Xanthobacteraceae and Hyphomicrobiaceae, and the genera *Bradyrhizobium* and *Haliangium*, etc. At the family level, Rhizobiaceae, Oxalobacteraceae, and Acidobacteriaceae_Subgroup_1 were notably enriched in the spring, autumn, and winter samples, respectively. Meanwhile, *Rhizobium*, *Herbaspirillum*, and *Burkholderia* were the genus-level biomarkers in the samples of those three seasons, in that order (Figure 5A). For fungi, fewer biomarkers were significantly enriched across the four seasons, with only 14 fungal groups identified at different taxonomic levels. These included two features in the summer sample (Chytridiomycota and Ceratobasidiaceae), two features in the autumn sample (Cladosporiaceae and *Cladosporium*), and one feature (Capnodiales) in the winter sample (Figure 5B).

### 3.4. Root Endophytic Microbial Function Prediction

**Figure 6:**
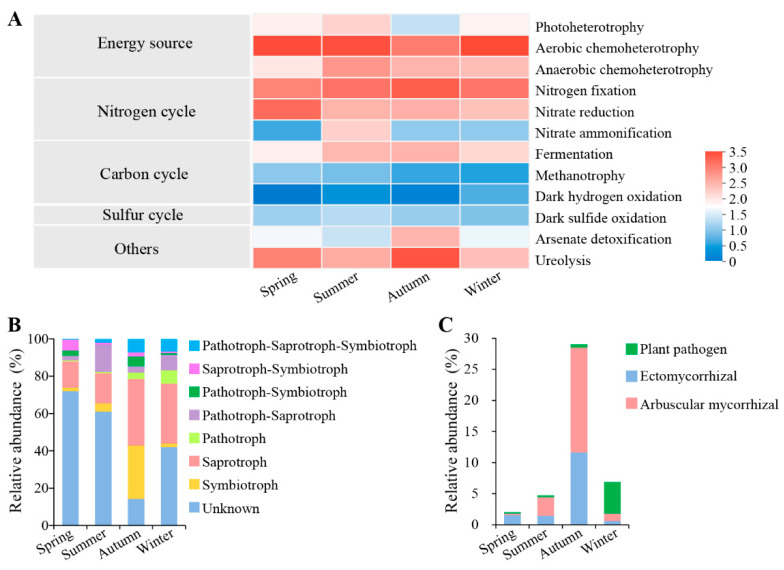
Functional prediction of endophytic microbes in root samples of *C*. *pingbianense* in different seasons. (**A**) The ecological function of endophytic bacterial communities predicted by FAPROTAX. (**B**) Fungal functional group composition of the samples. (**C**) Relative abundances of ectomycorrhiza, arbuscular mycorrhiza, and plant pathogen in samples of different seasons.

Based on the FAPROTAX database, we predicted the trophic types of root endophytic bacteria and the functional abundance of carbon, nitrogen, and sulfur cycles in which they may participate. The results (Figure 6A) showed that the main mode of energy acquisition by bacteria in roots was aerobic heterotrophy. The function of nitrogen fixation was found in high abundance in all samples, with the highest levels being found in the autumn sample. The relative abundance of nitrate reduction was highest in the spring sample. Nitrate ammonification was less abundant compared to the previous two functions and was characterized by the highest abundance in the summer sample. The abundance of the fermentation function was relatively high in the summer and autumn samples. Across different seasons, some bacteria may participate in the transformation between different valence states of sulfur compounds, but their abundance was low. In addition, functions related to arsenate detoxification and ureolysis were evidently more abundant in the autumn sample compared to the other seasons.

The functional guild composition assigned by FUNGuild revealed that the proportion of symbiotrophic fungi in the autumn sample (28.59%) was clearly higher than in the other three seasons. These fungi were mainly ectomycorrhiza (e.g., *Russula*, *Lactarius*, and *Chloridium*) and arbuscular mycorrhiza (e.g., *Acaulospora*, *Dominikia*, and *Archaeospora*). The relative abundances of saprotrophic fungi in the autumn (35.67%) and winter samples (32.20%) were higher than in the spring (14.13%) and summer (16.14%) samples. These primarily included undefined saprotrophs (e.g., *Trichoderma*) and soil saprotrophs (e.g., *Apiotrichum*). Pathotrophic fungi demonstrated the highest relative abundance (7.15%) in the winter sample, primarily consisting of plant pathogens (e.g., *Devriesia*) and animal pathogens (e.g., *Pochonia*) (Figure 6B). Furthermore, we compared the relative abundances of ectomycorrhiza, arbuscular mycorrhiza, and plant pathogens in samples from all seasons. As shown in Figure 6C, the relative abundances of the first two were much higher in the autumn sample than in the other seasons, while the proportion of plant pathogens peaked in the winter sample.

### 3.5. Correlation between Bamboo Shoot Number and Root Endophytic Microorganism

**Figure 7:**
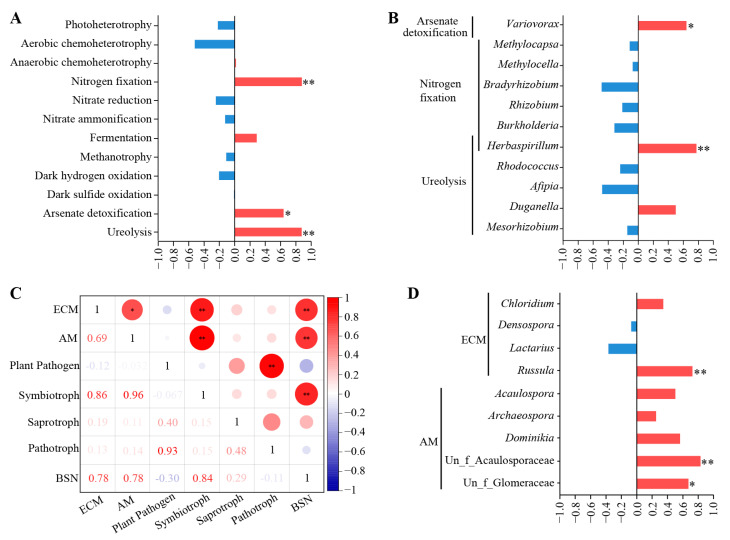
Correlation analysis between bamboo shoot number and endophytic microbes in root samples of *C*. *pingbianense*. (**A**) Correlations between bamboo shoot number and important bacterial functions. (**B**) Correlations between bamboo shoot number and the bacterial genera with important functions. (**C**) Correlations between bamboo shoot number and important fungal funguild. (**D**) Correlations between bamboo shoot number and the fungal genera belonging to ectomycorrhiza and arbuscular mycorrhiza. ECM, ectomycorrhizal fungi; AM, arbuscular mycorrhizal fungi. * and ** indicate significant differences at the *p* < 0.05 and *p* < 0.01 levels, respectively.

By analyzing the correlations between bamboo shoot number (Table S1) and the main functions of root endophytic bacteria in *C. pingbianense*, we found significant or extremely significant positive correlations between shoot production and the abundance of nitrogen fixation (r = 0.874, *p* < 0.01), ureolysis (r = 0.878, *p* < 0.01), and arsenate detoxification (r = 0.640, *p* < 0.05) (Figure 7A). The major bacterial genera associated with these functions were further screened and analyzed for their correlation with shoot production. The results showed that *Variovorax*, involved in arsenate detoxification, was significantly positively correlated with bamboo shoot number (r = 0.640, *p* < 0.05), while *Herbaspirillum*, involved in nitrogen fixation and ureolysis, had a highly significant positive correlation with the shoot number (r = 0.773, *p* < 0.01) (Figure 7B). 

The correlations between bamboo shoot number and the main functional guilds of root endophytic fungi were also analyzed. What we obtained was that the abundance of plant pathogens was negatively correlated with the bamboo shoot number. Conversely, there was a highly significant positive correlation between bamboo shoot number and symbiotrophic fungi (r = 0.839, *p* < 0.01), ectomycorrhiza, (r = 0.783, *p* < 0.01), and arbuscular mycorrhiza (r = 0.776, *p* < 0.01) (Figure 7C). Further, the major fungal genera belonging to ectomycorrhiza and arbuscular mycorrhiza were screened, and their correlations with shoot production were analyzed. The results showed that *Russula* (r = 0.725, *p* < 0.01), belonging to ectomycorrhiza, and unclassified_f_Acaulosporaceae (r = 0.831, *p* < 0.01) and unclassified_f_Glomeraceae (r = 0.672, *p* < 0.05), belonging to arbuscular mycorrhiza, were significantly correlated with shoot production (Figure 7D).

## 4. Discussion

### 4.1. The Seasonal Variations in the Bacterial and Fungal Communities in C. pingbianense Roots May Be Influenced by Temperature and Precipitation

The endophytic community is highly dynamic, with its colonization and growth influenced by various factors [37,38,39]. Our experimental results indicate that season is one of the driving factors affecting the community diversity and structure of root endophytic microbes of *C. pingbianense*. Seasonal variation of an endophytic community may be influenced by changes in soluble sugars, proteins, and other nutrients in the host plant. For example, a study on hybrid bamboo *Bambusa pervariabilis* × *Dendrocalamopsis grandis* showed that potassium tissue content is an important factor linked with the observed seasonal changes in the bamboo root microbiome [40]. In addition, climate, especially temperature and precipitation, can also affect the diversity and composition of the endophytic community [41,42]. The root endophytic bacterial community of *C. pingbianense* exhibited the highest alpha diversity in the summer, which may be affected by the high temperature. Similar to this result, the colonization of endophytic bacteria in elm and mulberry increased during the warm season [17,43], and the richness and diversity indexes of the root endophytic bacterial community in *Festuca sinensis* showed a significant positive correlation with the annual average temperature [44]. Generally, the diversity of endophytic fungi in the rainy season is higher than in the dry season, as observed in mangroves [45], wild grapes [46], and lowland forests [47]. Therefore, the higher endophytic fungal diversity in the summer and autumn samples may be attributed to the increased rainfall during this period. Furthermore, the enrichment of ectomycorrhizal and arbuscular mycorrhizal fungi in the autumn sample may have contributed to the relatively high diversity of the fungal community at that time.

### 4.2. The Abundant Nitrogen-Fixing Bacteria and Mycorrhizal Fungi in C. pingbianense Roots May Enhance Nutrient Absorption and Utilization

Studies have shown that plant endophytic bacteria are typically enriched with members of Proteobacteria and Firmicutes, with their relative abundance being at least twice as high as in the rhizosphere [48]. In this study, the phylum Proteobacteria was the dominant bacterial taxon in the roots of *C. pingbianense*. Compared to rhizosphere bacteria, the root endophytic bacterial community of *C. pingbianense* had a higher proportion of Proteobacteria and Firmicutes [27], which was consistent with previous studies [10]. Bacterial taxa of Burkholderiales and Rhizobiales were present in high abundance in samples from all seasons. At the genus level, *Burkholderia* occupied a dominant position. *Burkholderia* exhibits various functions, with some being animal pathogens and others contributing to various important environmental processes, including the nitrogen cycle. In some gramineous plants such as sugarcane [49], maize [50], and rice [51,52], *Burkholderia* has been documented as a nitrogen-fixing endophyte and has a role in promoting plant growth. Predicted bacterial functions showed a high abundance of nitrogen fixation for bacterial communities in all seasons. However, except for *Burkholderia*, the relative abundances of other main nitrogen-fixing bacteria such as *Rhizobium* and *Herbaspirillum* varied across seasons. At the family level, biomarkers related to nitrogen fixation were significantly enriched in different seasons, such as Rhizobiaceae in spring, Xanthobacteraceae and Hyphomicrobiaceae in summer, and Oxalobacteraceae in autumn. Among them, Rhizobiaceae, Xanthobacteraceae, and Hyphomicrobiaceae belong to the same order of Rhizobiales, while the Oxalobacteraceae taxa have been shown to play a role in promoting plant growth and nitrogen absorption [53]. This suggests that the roots of *C. pingbianense* may facilitate nitrogen fixation through different endophytic nitrogen-fixing bacterial groups in different seasons.

In terms of endophytic fungi, the spring and summer samples contained large amounts of taxa belonging to Basidiomycota, with a high proportion of unidentified Agaricomycetes, resulting in a high percentage of fungi with unknown trophic types during functional analysis. In another way, it suggested that there may be numerous new fungal groups in the roots of *C*. *pingbianense*, with the potential for further development. At the genus level, *Trichoderma* (belonging to Hypocreales), *Russula* (belonging to Russulales), and unclassified_f_Glomeraceae (belonging to Glomerales) exhibited high relative abundance in the autumn sample. Among them, *Trichoderma* is an important antagonist of plant pathogens, capable of inhibiting pathogenic fungi by secreting cell wall degrading enzymes and antifungal secondary metabolites [54,55]. In addition, it can release substances similar to auxin, gibberellin, or cytokinin to promote plant growth and productivity [56]. Members of Russulales and Glomerales often form reciprocal mycorrhizal structures with plant roots, where mycorrhizal fungi obtain carbohydrates from the host plant and, in turn, provide mineral nutrients such as phosphorus or nitrogen to the plant [57]. Arbuscular mycorrhizas and ectomycorrhizas are the main mycorrhizal types, with the former being more widely distributed. Arbuscular mycorrhizal fungi are symbiotic with more than two-thirds of terrestrial plants, and their hyphae can transfer phosphorus from the soil to the plant by entering the interior of the plant root cells [58]. The hyphae of ectomycorrhizas can infest the lower epidermal cells of newborn root tips and transfer soil nitrogen to the roots [57,59]. In this study, the relative abundances of arbuscular mycorrhizas and ectomycorrhizas were visibly higher in the autumn sample than in other seasons, which may help produce more bamboo shoots in the fall. Additionally, in the case of the soil phosphorus deficiency in autumn [27], roots may obtain phosphorus more effectively through the indirect phosphate uptake pathway via symbiosis with arbuscular mycorrhizas. Due to the similarity in chemical properties between arsenate (H_2_AsO_4_^−^) and phosphate (H_2_PO_4_^−^), arsenate can enter root cells via the phosphate uptake pathway and be reduced to arsenite [60]. Along with phosphorus acquisition, plants may absorb a high amount of arsenate, thus eliciting a detoxification response in cells. This may account for the enrichment of the arsenate detoxification function in the bacterial community in the autumn sample.

### 4.3. Root Endophytic Microorganisms Have a Positive Impact on the Year-Round Bamboo Shooting Characteristic of C. pingbianense

Plant endophytes have been shown to have great potential in resisting biological stress and improving crop productivity. Our study revealed that the bamboo shoot number of *C. pingbianense* was significantly and positively correlated with the relative abundance of *Herbaspirillum*, *Russula*, and certain taxa belonging to Glomeraceae and Acaulosporaceae. Among these, *Herbaspirillum* is one of the common endophytic nitrogen-fixing bacteria, which can be found in a variety of gramineous plants such as rice, maize, and sugarcane [61,62]. It has been shown that inoculation with endophytic *Herbaspirillum* in rice seedlings can promote their growth, nutrient absorption, and photosynthetic efficiency [63]. After inoculation with *Herbaspirillum*, the nitrogen uptake and assimilation of maize seedlings increased in the early developmental stages, along with an increase in both aboveground and underground biomass [64]. The fungal genus *Russula*, a representative species of Russulaceae, can form ectomycorrhizas with a variety of plants and can also develop different types of mycorrhizal interactions [57]. Arbuscular mycorrhizas are considered to be non-specific symbiotic fungi, with low host specificity which increases the likelihood of fungal hyphae connecting multiple plants to form common mycelial networks [65,66]. The mycelial networks can connect host and non-host plants, facilitating the transfer of nutrients such as carbon, nitrogen, and phosphorus among different plant species. Bacterial or fungal taxa such as Glomeraceae, Acaulosporaceae, *Herbaspirillum*, and *Russula* may positively influence the productivity of *C. pingbianense* by enhancing the uptake of nutrients through the roots. 

## 5. Conclusions

This paper focused on the seasonal variation of root endophytic microbial communities of *C. pingbianense*, the functional potential of key microbial taxa, and their effect on shoot productivity. The information we obtained was that the bacterial communities in samples from different seasons contained a high number of nitrogen-fixing microorganisms, most of which were members of Burkholderiales and Rhizobiales. The functions of nitrogen fixation, arsenate detoxification, and ureolysis in bacterial communities, along with the key microbial groups associated with these functions, were significantly and positively correlated with the bamboo shoot number of *C. pingbianense.* The relative abundances of ectomycorrhizal and arbuscular mycorrhizal fungi were also significantly positively correlated with bamboo shoot numbers and were notably higher in the autumn sample compared to the other seasons. 

We believe that root endophytic microorganisms may positively influence the shoot productivity of *C. pingbianense* by increasing the availability or supply of nutrients. This study provides a scientific basis for understanding the year-round shooting characteristic of *C. pingbianense*. It also serves as a scientific reference for the targeted isolation and pure culture of specific key microbial groups to regulate the microbial community and thus improve the growth of bamboo shoots.

## Data Availability

The raw data supporting the conclusions of this article will be made available by the authors on request.

## Supplementary Materials

The following are available online at https://www.mdpi.com/article/10.3390/microorganisms12091927/s1, Table S1: Bamboo shoot number of *C. pingbianense* in the four seasons, Table S2: Sequencing information of endophytic microbial communities in root samples of *C. pingbianense* in the four seasons.
